# Effectiveness of treatment with high-frequency chest wall oscillation in patients with bronchiectasis

**DOI:** 10.1186/1471-2466-13-21

**Published:** 2013-04-04

**Authors:** Antonello Nicolini, Federica Cardini, Norma Landucci, Sergio Lanata, Maura Ferrari-Bravo, Cornelius Barlascini

**Affiliations:** 1 Respiratory Diseases Unit,General Hospital of Sestri Levante, Genoa, Italy; 2 Histopathology Unit, General Hospital of Sestri Levante, Genoa, Italy; 3 Department of Public Health and Preventive Medicine, Chiavari, Italy; 4 Forensic Medicine, Chiavari, Italy

**Keywords:** Bronchiectasis, High frequency chest wall oscillation, Chest physiotherapy, Lung function, Sputum cell count, Dyspnea scales

## Abstract

**Background:**

High-frequency airway clearance (HFCWC) assist devices generate either positive or negative trans-respiratory pressure excursions to produce high-frequency, small-volume oscillations in the airways.

HFCWC can lead to changes in volume of 15–57 ml and in flow up to 1.6 L/s, which generate minimal coughing to mobilize secretions. The typical treatment lasts 20–30 minutes, and consists of short periods of compression at different frequencies, separated by coughing.

The aim of this study was to find the more efficacious treatment in patients with bronchiectasis: traditional techniques of chest physiotherapy (CPT) versus high frequency oscillation of the chest wall in patients with bronchiectasis.

**Methods:**

37 patients were enrolled. Seven of them were excluded. Computer randomization divided the patients into three groups:

– 10 patients treated with HFCWO by using the Vest® Airway Clearance System;

– 10 patients treated with traditional techniques of air way clearance (PEP bottle, PEP mask, ELTGOL, vibratory positive expiratory pressure);

– 10 patients received medical therapy only (control group).

To be eligible for enrollment, participants had to be between 18 and 85 years old and have a diagnosis of bronchiectasis, confirmed on high resolution computed tomography. Exclusion criteria: lack of informed consent, signs of exacerbation, cystic fibrosis. Before the treatment, each patient had blood tests, sputum volume and cell count, pulmonary function tests and on the quality of life inventories (MMRC, CAT, BCSS). The results were processed through the covariance analysis, performed with the R-Project statistical program. It has been considered a positive result p <005.

**Results:**

Both treatments (traditional CPT and HFCWO) showed a significant improvement in some biochemical and functional respiratory tests as well as in the quality of life compared to the control group. The use of HFCWO compared to CPT also produced a significant improvement in blood inflammation parameter C-RP (p ≤0.019), parameters of lung functionality associated with bronchial obstruction (FVC, FEV1) (p ≤0.006 and p ≤0.001), and in the dyspnea. Improvement in quality of life scales was noted. (BCSS, CAT) (both p ≤0.001). No significant changes of total cell counts in sputum samples were observed in the two groups. In the HFCWO group a significant reduction of neutrophils percentage (p≤0.002) and a significant increase of macrophages percentage (p ≤0.012).

**Conclusions:**

The HFCWO technique provides an improvement both in pulmonary function and quality of life related parameters in patients with chronic hypersecretive disease. Since those patients need daily airway clearance, this treatment should be included among the principal options in chest physiotherapy. The study was registered as ChiCTR-TRC-12002134 at http://www.chictr.org.

## Background

Bronchiectasis is defined as an irreversible dilatation and destruction of the bronchi [1] with a reduction in clearance of secrections (and particularly in the expiratory airflow) [2]. This disease can lead to recurrent lower respiratory tract infections and worsening pulmonary function, with increased morbidity and mortality [2-5]. The incidence and the prevalence of bronchiectasis is not known, but its diagnosis has increased mainly due to the more frequent use of high-resolution computed tomography [2,6]. Bronchiectasis is usually associated with chronic cough, increasing secretions, and recurrent airway and pulmonary infections [6]. The fundamental aspects in these patients are the colonizations and infection of the bronchial mucous by potentially pathogenic microorganisms such as Pseudomonas Aeruginosa, Burkholderia cepacia and others. This chronic process results in the destruction and dilatation of the bronchial tree that is the characteristic of the disease [7]. The goals of bronchiectasis treatment are to reduce the number of exacerbations and infections and to improve patient quality of life by reducing airway inflammation and mobilizing secretions [6,8,9]. Therapies showing to be effective in cystic fibrosis are often provided to patients with bronchiectasis,without definitive evidence of benefit. In recent years, there has been increased interest in validating and developing new therapies for patients without cystic fibrosis [10]. These include inhaled antibiotics (tobramycin, aztreonam, ciprofloxacin, colistin, amikacin) [10], hyperosmolar agents (hypertonic saline solution, dry powder mannitol) [10,11], anti-inflammatory agents (macrolides, corticosteroids) [7,12], bronchodilators (salbutamol) [13],chest physiotherapy, physical exercise and nutritional treatment [7,14-16]. In the field of chest physiotherapy several secretion management techniques have been proposed: they include modified postural drainage [17], assisted cough [17], active cycle of breathing techniques [17,18], oscillatory positive-expiratory pressure devices [17,18] and intrapulmonary percussive ventilation [6]. Although the mucous clearance is recommended in bronchiectasis, there are no definitive studies or guidelines on the preference or superiority of one technique versus the others [6,19]. High frequency chest wall oscillation (HFCWO) is widely used in the USA where is considered standard care in cystic fibrosis (CF) [20,21]. It has recently been introduced to UK and Europe and has been used in several other pulmonary diseases, different from CF like chronic obstructive pulmonary disease [22] or exacerbations of chronic obstructive pulmonary disease or bronchial asthma [23]. To our knowledge there have been no trials of HFCWO in patients with non-CF bronchiectasis. The aim of the study was to compare the efficacy, the safety of HFCWO with our standard traditional chest physiotherapy (CPT) in patients with non-CF bronchiectasis.

## Methods

37 Adults (aged 18 years and older) with a chest computed tomography confirmed diagnosis of bronchiectasis were admitted to the study in the Respiratory Disease Unit of General Hospital of Sestri Levante,Italy from April to June 2012. The inclusion criteria were:

– Daily sputum volume ≥ 20 ml daily at least 3 consecutive days [22]

– Clinical stability: no need for medication changes a week prior to enrollment

– Normal gas exchange: ph ≥7.35 during spontaneous breathing, with or without supplemental oxygen

– No major cardiac arrhythmias or hemodynamic instability.

The exclusion criteria were cystic fibrosis, tracheostomy, non-invasive ventilation, inability to perform forced expiratory maneuvers, recent episode of significant hemoptysis, or pneumothorax in the six months preceding enrollment. The drop-out criteria were withdrawal of patient consent, severe clinical worsening, chest radiograph changes and occurrence of any of the exclusion criteria.

The study was carried out according to the rules of the declaration of Helsinki and approved by Ethics committee of ASL 4 Chiavarese all patients provided written informed consent before beginning the study. The study was registered as Chi CTR-TRC-12002134 at http://www.chictr.org.

### Protocol

**Figure 1:**
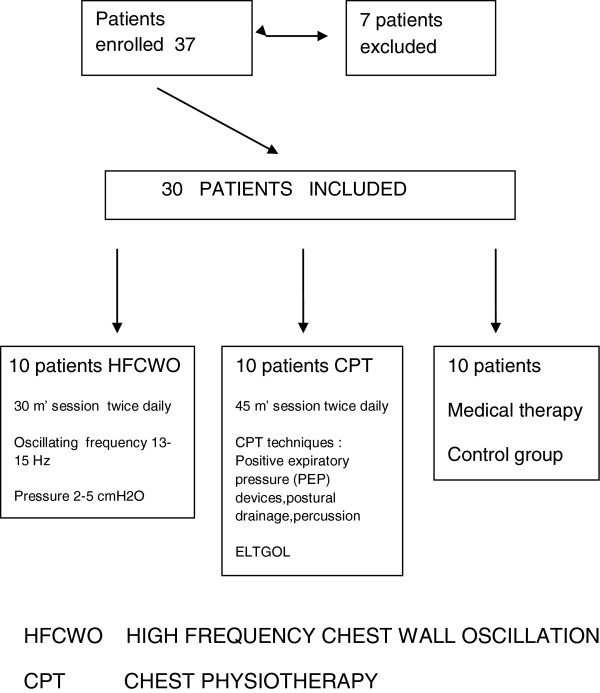
Patients flow.

Every patient was assigned following a computed randomized list to High frequency chest wall oscillation (HFCWO) or to chest physiotherapy (CPT) or to medical therapy only (control group) (Figure 1). CPT secretion clearance sessions lasted 45 minutes per session; HFCWO lasted 30 minutes per session. Both treatments were given twice daily (morning and late afternoon).The duration of each treatment was fifteen days: the treatment was administered five days per week. High frequency chest wall oscillation (HFCWO). HFCWO was provided with the Vest® Airway Clearance System (Hill-Rom, Batesville, Indiana, USA).The Vest system consists of an inflatable vest, which is worn over the torso and an air pulse generator that delivers the oscillating air pulses to the vest via a connecting air hose. The patient was in upright sitting position and the Vest air pulse generator was set to an optimum oscillating frequency of 13–15 Hz based on individual patient tolerance and a pressure setting of 2–5 cm H2O to achieve a tight but comfortable snug fit [22]. Every session lasted 30 minutes and every patient had a treatment twice per day (morning and late afternoon). Chest Physiotherapy (CPT) consisted of a group of respiratory physiotherapy techniques like, slow expiratory with glottis opened in lateral position (ELTGOL) [24], positive expiratory pressure (PEP) mask [25] or PEP bottle [26] and vibratory positive expiratory pressure therapy system [27] (Acapella choice, Smiths Medical, England). Every session lasted 45 minutes and every patient had a treatment twice per day (morning and late afternoon).

### Measurements

Primary outcome measures included dyspnea, cough, and sputum scales, as well as daily life activity evaluations. Secondary outcome measures were respiratory function testing and, hematological examinations, and sputum cell count. Dyspnea, cough and sputum and daily life activity was measured with the Breathlessness, Cough, and Sputum Scale (BCSS) [28], COPD Assessment Test (CAT) [29,30] and Modified Medical Research Council (MMRC) Dyspnea Scale. Sputum sample for analysis was defined as that containing expectorated material with cellular viability greater than 50% and contamination by oropharyngeal squamous cell cells lower than 20%, as well as being of a quantity sufficient for differential counts of 400 cells [31,32]. Sputum collection was made the day of the starting of the treatment and the day of the last treatment. The patients were instructed by the physiotherapists or by the nurses to expectorate into the sputum cups during the entire duration of the treatment and to continue expectoring if the patient felt the need to cough. All sputum produced over the 60-minute period was collected [33]. Pulmonary function testing was performed with a computerized body plethysmography (VMAX 20 PFT Sensor Medics, Yorba Linda, CA,US), according to the international standards [34]. The baseline characteristics of the patients are shown in Table 1.

**Table 1:** Baseline characteristics of the patients

	**Control group**		**HFWCO group**		**CPT group**	
**SEX (Male/Female)**	**4 male 6 female**		**3 male 7 female**		**2 male 8 female**	
	**Mean**	***SD***	**Mean**	***SD***	**Mean**	***SD***
AGE(years)	71.9	*7.46*	74.6	*4.69*	73.9	*3.66*
FVC°(ml)	2125	*898.9*	2545	*820*	2427.5	*813.7*
FEV1°°(ml)	1016	*628.9*	1509	*625.5*	1739	*672.9*
Tiffeneau Index	46.5	*14.1*	58.4	*13.8*	68.7	*13.3*
TLC°°°(ml)	5593	*2013*	5993	*2407.2*	5931	*1546.8*
RV°°°°(ml)	3423	*2047.4*	3161	*1106.5*	3353	*2320.6*
paO2(mmHg)	66.2	*4.9*	70.9	*8.6*	76.3	*12.3*
paCO2(mmHg)	42.4	*1.4*	42.5	*4.6*	37.4	*6.6*
MIP*(cmH2O)	50.5	*10.4*	61	*13*	54.8	*16.7*
MEP**(cmH2O)	65	*12.6*	66.6	*17.7*	65	*23.2*
MMRC***	2.2	*0.4*	2.1	*0.7*	2.3	*1.3*
CAT****	17.9	*8.5*	23.9	*6.3*	17.7	*8.3*
BCSS*****	6.2	*2.4*	6.8	*2.8*	4.9	*2.8*

°Forced vital capacity * Maximal Inspiratory Pressure.

°°Forced expiratory volume 1 minute ** Maximal Expiratory Pressure.

°°° Total lung capacity *** Modified Medical Research Scale.

°°°° Residual volume **** COPD Assessment Test.

***** Breathlessness, Cough and Sputum Scale.

### Statistical analysis

Clinical data were expressed as count and mean and standard deviation. We calculated the difference between the two treatments (HFCWO and CPT) and control group using univariate (covariance) analysis. Subsequently, the difference between the two treatments (HFCWO and CPT) was analyzed using covariance analysis; p≤0.05 was considered statistically significant. Data analysis was made with statistics software R-Project version 2.13.2.

## Results

### Participants

There were 52 patients admitted during the study period: 37 patients were screened and 30 were enrolled (9 men and 21 women) (Table 1). The reasons for exclusion were: recent episode of significant hemoptysis (1 patient), episode of pneumothorax in the six months preceding enrollment (1 patient), inability to perform forced expiratory maneuvers (2 patients), refusal (3 patients). All the 20 patients (7 males and 13 females) assigned to airway clearance sessions completed their sessions. None of the patients enrolled withdrew from the study because of discomfort with HFCWO device or CPT. None had exacerbations. All were clinically stable and able to cough spontaneously. Moreover, the patients in the control group did not have exacerbations: in three cases they presented an average increase in sputum volume of 10 ml at the end of the study.

#### Measurements

**Figure 2:**
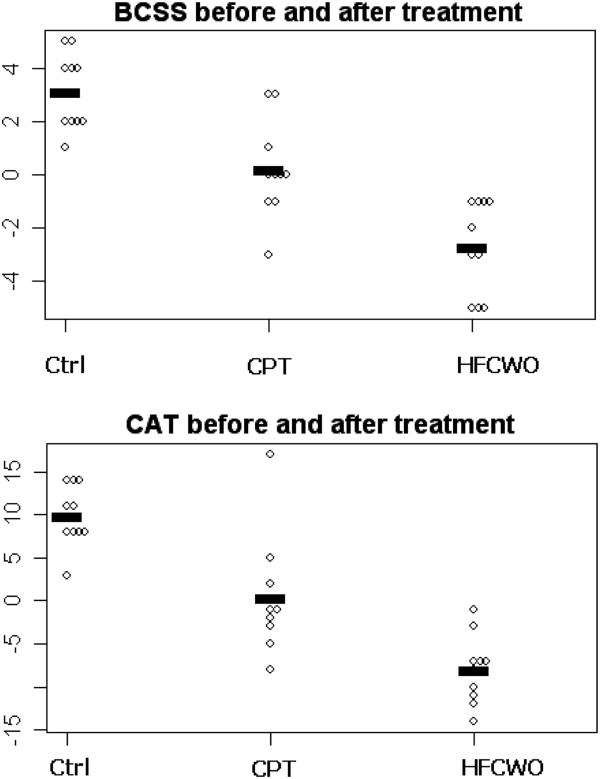
Dot-plot that shows BCSS score and CAT score change-values before and after treatment in HFCWO and CPT groups.

**Breathlessnes and life quality scales** Both treatment showed an increase in all the three test used for the assessment of dyspnea and quality of life (BCSS, MMRC, CAT) respect to control group. HFCWO showed a significant improvement in BCSS (p ≤0.001) and CAT (p ≤0.001) than CPT (Figure 2).

**Figure 3:**
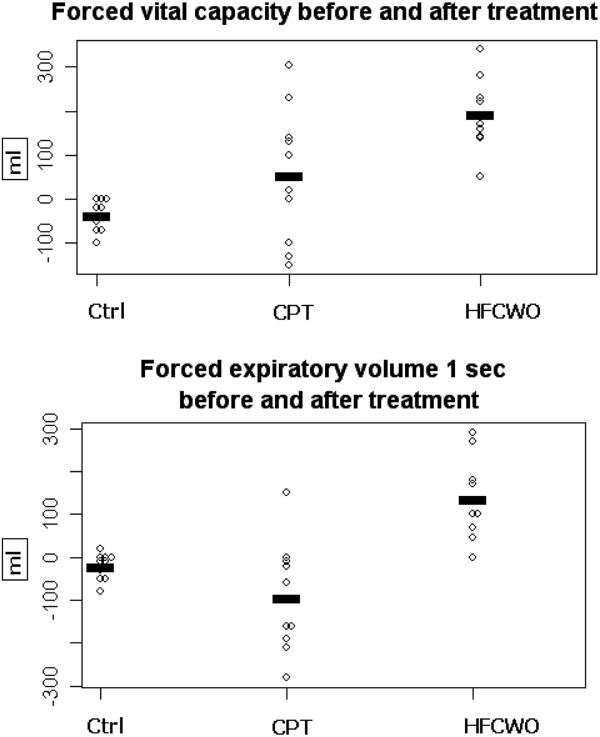
Dop-plot that shows FVC and FEV1 change values (ml) before and after treatment in HFCWO and CPT groups.

**Respiratory function and laboratory measurements** Both groups (CPT and HFCWO) presented a significant improvement in pulmonary function tests (FVC and FEV1) in comparison with control group. Moreover, the HFCWO group showed a significant increasing of FVC and FEV1 after treatment (p ≤0.006 and p≤0.001) (Figure 3). Among biochemical laboratory blood measurements the HFCWO group showed a significant reduction of C-reactive protein compared to CPT group (p ≤0.019).

**Sputum volume** Both HFCWO and CPT increased after treatment the sputum production: from 62.5±18.9 ml at admission to 70.0± 21.1 in the CPT group and from 52.0±16.9 ml to 72.5 ± 24.0 ml in the HFCWO group. There was a significant difference in HFCWO group, where the treatment produced a greater increase of sputum volume at the end of treatment (p≥ 0.011). All the results of the measurements are reported in Table 2. The table displays covariate-unadjusted mean levels.

**Table 2:** Biochemical, lung function, and QOL value before and after in the three groups

	**Control group (Ctrl)**		**CPT treatment**		**HFCWO treatment**		**Difference between after and before**		
	**difference**		**difference**		**difference**		***CPT***	***HFCWO***	***HFCWO***
	***After-before***		***After-before***		***After-before***		***vs Ctrl***	***vs Ctrl***	***vs CPT***
	**Mean**	***SD***	**Mean**	***SD***	**Mean**	***SD***	***P-value (covariance analysis)***		
WC (103 cell)	957.0	915.7	407.0	2211.2	−673.8	1093.6	0.42	**0.02**	0.123
RC (106 cell)	−82.0	62.3	26.0	164.7	73.0	202.5	0.13	**0.03**	0.503
Neutr %	5.4	9.2	1.2	4.2	−4.0	8.5	0.23	**0.01**	0.136
Lymph %	−1.4	3.7	−1.4	2.8	0.6	7.2	0.99	0.37	0.377
FVC (ml)	−37.0	35.0	54.5	153.7	192.1	80.9	0.06	**0.001**	**0.006**
FEV1 (ml)	−21.0	30.7	−94.0	128.3	135.5	93.4	0.09	**0.001**	**0.001**
Tiff.Ind.	−0.6	1.3	−0.6	3.5	3.1	6.7	0.99	0.07	0.072
TLC (ml)	46.0	95.6	−88.0	312.4	−657.0	1088.9	0.65	**0.02**	0.063
RV (ml)	65.0	58.5	−145.0	327.8	−580.0	1118.1	0.49	**0.04**	0.160
Mip (cmH2O)	−4.1	2.5	2.1	13.2	9.8	10.1	0.16	**0.003**	0.088
Mep (cmH2O)	−8.3	3.9	2.4	23.6	6.5	7.2	0.10	**0.03**	0.530
paO2 (mmHg)	−1.4	3.5	0.8	9.5	1.7	8.8	0.54	0.38	0.786
paCO2 (mmHg)	0.9	1.8	1.1	2.5	−0.9	2.7	0.82	0.10	0.066
ph	−0.004	0.010	0.001	0.023	0.016	0.028	0.59	0.049	0.143
BCSS	3.1	1.4	0.2	1.8	−2.7	1.8	**0.001**	**0.001**	**0.001**
MMRC	1.0	0.8	−0.5	1.1	−0.7	0.8	**0.001**	**0.001**	0.629
CAT	9.9	3.6	0.4	6.8	−8.0	4.0	**0.001**	**0.001**	**0.001**
C-R Prot.	1.3	1.1	0.0	0.9	−1.0	0.8	**0.01**	**0.001**	**0.019**

The table displays covariate-unadjusted mean levels.

WC White cells - RC Red cells - FVC Forced vital capacity - FEV1 Forced Expiratory volume 1 sec - Tiff Ind Tiffeneau Index - TLC Total lung capacity - RV residual volume - MIP Maximal inspiratory pressure - MEP Maximal expiratory pressure.

BCSS Breathlessness, Cough, Sputum, Scale - MMRC Modified Medical Research Council Dyspnea Scale - CAT COPD assessment test -C-R prot C-reactive protein Ctrl Control group CPT Chest physiotherapy group HFCWO high frequency chest wall oscillation group.

QOL Quality of life.

**Sputum cellularity** No significant changes of total cell counts in sputum samples were observed in the two groups. In the HFCWO group a significant reduction of neutrophils percentage (p ≤0.002) and a significant increasing of macrophages percentage (p ≤0.012) was observed. All the results of the sputum cellularity measurements are reported in Table 3. The table displays covariate-unadjusted mean levels.

**Table 3:** Sputum cytological changes before and after in the three groups

	**Control group (Ctrl)**				**CPT treatment**				**HFCWO treatment**				**Difference between after and before**		
	**Before**		**After**		**Before**		**After**		**Before**		**After**		***CPT***	***HFCWO***	***HFCWO***
													***vs Ctrl***	***vs Ctrl***	***vs CPT***
	**Mean**	***SD***	**Mean**	***SD***	**Mean**	***SD***	**Mean**	***SD***	**Mean**	***SD***	**Mean**	***SD***	***P-value (covariance analysis)***		
TCCx 106/mg	9891	*1797.3*	10517	*2514.9*	9.636	*3.181*	8.490.	*2.771*	9.671	*2.136*	7.225	*1.186*	**0.001**	**0.001**	0.096
Neutroph %	70.54	*5.5*	78.09	*6.8*	65.3	*10.1*	62.0	*9.9*	72.5	*9.2*	59.9	*10.1*	**0.001**	**0.001**	**0.002**
Lymphoc %	9.11	*3.6*	7.17	*2.7*	11.3	*4.8*	13.5	*3.9*	10.2	*5.2*	11.9	*4.9*	**0.001**	**0.001**	0.548
Eosin %	1.09	*0.46*	0.97	*0.44*	0.9	*0.4*	0.7	*0.4*	0.6	*0.2*	0.6	*0.1*	0.7	0.5	0.333
Macroph %	37.2	*7.8*	32.2	*10.8*	26.9	*8.5*	31.2	*7.5*	19.9	*11.1*	35.6	*15.2*	**0.03**	**0.001**	**0.012**
Sputum (ml)	74.0	*10.7*	77.0	*10.6*	70.0	*21.1*	62.5	*18.9*	72.5	23.9	52.0	*16.9*	**0.04**	**0.001**	**0.01**

TCC Total cell count.

Neu Neutrophyls.

Lymph Lymphocytes.

Eos Eosinophyls.

Macr Macrophages.

CPT Chest Physiotherapy.

HFCWO High frequency chest wall oscillation.

Ctrl Control group.

## Discussion

Techniques for augmenting the normal muco-ciliary and cough clearance mechanisms of the lungs are not new, but in recent years several techniques have been developed which are effective and comfortable; these techniques can be used without an assistant in the majority of adolescents and adults [35]. HFCWO has been one of the most studied techniques in the more recent years and it has been used in many circumstances such as thoracic trauma [36], neuromuscular diseases, chronic obstructive pulmonary disease [22,23,37,38], bronchial asthma [23], and cystic fibrosis [38-47]. In chronic obstructive pulmonary diseases HFCWO produces improvements in gas mixing and homogenization of alveolar ventilation for previously closed or under ventilated lung units [37,44]. HFCWO has been shown to decrease functional residual capacity (FRC) in subject with obstructive lung disease [37,39]:this could explain the improvement of FVC we have observed. Moreover, high frequency chest wall oscillation delivers an intermittent flow of air into the jacket which rapidly compresses and releases the chest wall at a variety of frequencies. An oscillation in airflow within the airways is achieved. HFCWO has been shown to augment central and peripheral mucus clearance [21]. Only one study reported an improvement in FEV1 in the longer term using [46], and few trials have compared HFCWO with traditional chest physiotherapy in cystic fibrotic (CF) patients with favorable results for HFCWO [21,41,46]. The effects of HFCWO on sputum production as well as lung function is in dispute. Compared with PEP, there was no difference in either lung function or sputum production [41,47]. When compared with oscillating PEP, one study showed a benefit in terms of sputum production but did not show differences in lung function [34,41,48]. A recently published study on hypersecretive COPD with recurrent exacerbations showed that the treatment with HFCWO led to improvement in lung function, quality of life, and reduction of symptoms, but not in sputum production [22]. An important limitation of most HFCWO studies is the short number of days of treatment. This makes it difficult to evaluate some outcomes like lung function or quality of life, which need much more time to change. None of the previous studies investigated sputum cellularity and its changes after airways clearance treatment. If we consider already published definitions used in the analysis of sputum cellularity (32, 33, 49) all the patients enrolled in the two groups (HFCWO and CPT) presented at starting a total cell count suggesting in all the patient the absence of infection. The reduction of percentage of neutrophils and the increase of percentage of macrophage could suggest a modulation of HFCWO in inflammatory cells (greater than CPT), but these data must be validated with further studies. The significance of this observation is not known.

### Limitations

We are aware of the limitations of our study. The amount of daily sputum volume was not reported; we collected sputum at before the first treatment and at the end of the last (when also performed cytological counts). We do not have concerning information about the daily variations in sputum volume. Moreover, this medium-term study does not allows us to provide information about the efficacy and acceptability of the device in the long-term.

## Conclusions

Our study showed that HFCWO produced an improvement in several lung function parameters compared to traditional chest physiotherapy. Long-term study are needed, not only to establish the effectiveness of different airways clearance devices or techniques and their cost-effectiveness, but especially to establish their acceptability in order to long-term home use.

## Abbreviations

HFCWO: High frequency chest wall oscillation; PEP: Positive expiratory pressure; ELTGOL: Slow expiration with glottis opened in lateral position; MMRC: Modified medical research council dyspnea scale; CAT: COPD assessment scale; BCSS: Breathlessness, cough and sputum scale; CPT: Chest physiotherapy; C-RP: C reactive protein; FVC: Forced vital capacity; FEV1: Forced expiratory volume 1 second; TLC: Total lung capacity; RV: Residual volume; MIP: Maximal inspiratory pressure; MEP: Maximal expiratory pressure; TCC: Total cell count; Neu: Neutrophyls; Lymph: Lymphocytes; Eos: Eosinophyls; Mac: Macrophages

## Competing interests

The authors declare that they have no competing interests.

## Authors’ contributions

NA: designed the study, analyzed and interpreted the data, drafted and revised the manuscript. CF designed the study, analyzed and interpreted the data, drafted and revised the manuscript. LN: data collection, analyzed and interpreted data Lanata Sergio: data collection, analyzed and interpreted data FBM: statistical analysis BC: data collection, analyzed and interpreted data, revised the manuscript. All authors read and approved the final manuscript.

## Pre-publication history

The pre-publication history for this paper can be accessed here:

http://www.biomedcentral.com/1471-2466/13/21/prepub
